# Diagnostic value of sFlt‐1/PlGF‐1 ratio and plasma PROK1 for adverse pregnancy outcomes in women with hypertensive disease of pregnancy

**DOI:** 10.1002/kjm2.12907

**Published:** 2024-12-03

**Authors:** Ping Lv, Lin‐Fei Lu

**Affiliations:** ^1^ Department of Obstetrical Shengzhou People's Hospital (The First Affiliated Hospital of Zhejiang University Shengzhou Branch) Shengzhou City Zhejiang Province China

**Keywords:** hypertensive disease of pregnancy, placental growth factor‐1, preeclampsia, prokinin‐1, soluble fms like tyrosine kinase‐1

## Abstract

Hypertensive disease of pregnancy (HDP) is one of the most important causes of increased maternal mortality and perinatal complications during pregnancy. We investigated the pregnancy outcomes of 156 HDP patients (65 gestational hypertension [GH], 13 chronic hypertension [CH], 74 preeclampsia‐eclampsia [PE‐EC], and 4 superimposed on PE [CH with PE]). In patients with different types of HDP, levels of soluble fms like tyrosine kinase‐1 (sFlt‐1), placental growth factor (PlGF)‐1, and prokinin‐1 (PROK1) were measured and compared. The predictive efficacy of these indicators was evaluated using receiving operating characteristics curves and area under the curve. Results showed that the PE cohort had a higher sFlt‐1/PlGF ratio (46.12 [39.24, 68.85]) and PROK1 (498.84 [213.67, 678.30] pg/mL) level than the GH (sFlt‐1/PlGF, 32.3 [21.98, 58.00], PROK1 300.77[250.0, 345.29]pg/mL) and CH cohort (sFlt‐1/PlGF, 37.49 [32.68, 39.68], PROK1, 281.48 [229.25, 453.94]pg/mL). In the HDP cohort, 54 patients experienced adverse pregnancy events, and the sFlt‐1/PlGF ratio, PROK1, and the combined indicators (sFlt‐1/PlGF ratio and PROK1) were excellent predictors of adverse pregnancy events, especially for PE patients.

## 1 INTRODUCTION

Hypertensive disease of pregnancy (HDP) is the most common disorder of pregnancy and is the most important cause of maternal mortality and perinatal complications.^1^ HDP is divided into the following categories: gestational hypertension (GH), chronic hypertension (CH), preeclampsia (PE)‐eclampsia (EC), and PE superimposed on CH (CH with PE).^2^ The severity of potential adverse effects caused by HDP ranges from mild clinical and laboratory manifestations to life‐threatening conditions. Serious adverse maternal outcomes include seizures, hemorrhagic complications, multiorgan failure, operative delivery, and death, and perinatal complications include preterm labor, small for gestational age (SGA), fetal growth restriction (FGR), stillbirth, and neonatal intensive care unit.^3^, ^4^ Many international guidelines now advocate strict monitoring once diagnosed and, if necessary, early delivery.^5^, ^6^ While past studies have delved into the potential negative effects associated with HDP, most have focused on specific HDP (especially patients with PE) rather than looking at all HDP cohorts collectively.^7^, ^8^, ^9^ Research findings assessing the risks of diverse HDP types reveal restricted comparability due to the variability in pregnancy outcomes among these patients, influenced by the diversity of the population. Analyzing different HDP types within the same group is crucial to comprehend potential adverse effects in HDP patients.

In the vascular endothelial cell injury doctrine, the balance of pro‐angiogenic and anti‐angiogenic factors in the placenta and maternal circulation has been a hot topic of research in the past two decades. Soluble fms‐like tyrosine kinase‐1 (sFlt‐1) is an important antiangiogenic factor.^10^ The imbalance in placental angiogenesis, due to elevated sFlt‐1 and reduced PlGF, leads to inadequate trophoblast invasion into the endometrium, thereby hindering the reshaping of small uterine spiral arteries, culminating in ischemia and hypoxia in the placenta.^11^, ^12^, ^13^ This hypoxic state in the placenta leads to the release of antiangiogenic factors into the maternal circulation, resulting in alterations in maternal systemic endothelial function and consequent hypertension.^14^ The sFlt‐1/PlGF‐1 ratio has been shown to predict PE in most studies.^15^, ^16^ In a short‐term prognostic study to predict pregnant women with suspected PE, the critical value of 38 is able to rule out PE at 1 week with a negative predictive value of 99.3% and to rule out disease at 4 weeks with a negative predictive value of 36.7%.^17^ Furthermore, the critical value of sFlt‐1/PlGF demonstrates high sensitivity and specificity in identifying diseases and their negative outcomes.^18^, ^19^, ^20^

Prokineticin 1 (PROK1) is a relatively new mitogen that promotes angiogenesis under hypoxia and is highly expressed in human fetal membranes, controlling trophoblast invasion, proliferation, and survival.^21^ In addition, elevated serum PROK1 levels in early pregnancy predict PE and intrauterine growth retardation.^22^ In another study, elevated PROK1 levels in amniotic fluid at mid‐pregnancy predict adverse pregnancy outcomes.^23^

Most studies have focused on investigating the sFlt‐1/PlGF ratio in the diagnosis of patients with PE in relation to pregnancy outcomes in such patients. In addition, most studies investigating serum PROK1 have focused on its use in early pregnancy to predict EC. Therefore, there is a need to investigate the levels of these biomarkers in patients with different types of HDP and their efficacy in predicting adverse pregnancy. The aim of this study was first to analyze the associated adverse pregnancy events in patients with different types of HDP in a prospective cohort. Subsequently, the sFlt‐1/PlGF ratio and PROK1 were analyzed to evaluate the predictive efficacy of associated adverse pregnancy events in patients with different types of HDP.

## 2 MATERIALS AND METHODS

### 2.1 Patients

This single‐center cross‐sectional cohort study was conducted at Shengzhou People's Hospital. Women diagnosed with HDP from January 2022 to December 2023 were included. Inclusion criteria: (1) patients with a clear diagnosis of HDP according to the Diagnosis and treatment of hypertension and pre‐eclampsia in pregnancy: a clinical practice guideline in China (2020); (2) No medication with an effect on blood pressure in the 1 month prior to diagnosis of HDP; (3) patients who had a singleton pregnancy; (4) patients who gave birth in Shengzhou People's Hospital; and (5) patients who had no obvious fetal structural abnormality or chromosomal abnormality indicated by ultrasonography. Exclusion criteria: (1) Patients aged <18 years or ≥45 years; (2) patients who suffered from serious abnormalities of cardiac, renal, and hepatic functions; (3) patients who combined with other serious cardiovascular diseases; (4) patients with other pregnancy complications, such as gestational diabetes mellitus and intrahepatic cholestasis; (5) patients with secondary hypertension caused by immune diseases such renal diseases, systemic lupus erythematosus, antiphospholipid syndrome, and so forth; and (6) patients who combined with uterine fibroids, ovarian cyst, and blood diseases. A total of 156 patients were finally included in this study. This study was approved by the Shengzhou People's Hospital Institutional Review Board (No.202101SZ‐05), and consent was obtained from the patients or their families.

### 2.2 Diagnostic criteria and grouping

This study referred to the relevant criteria in Obstetrics and Gynecology (9th edition) to assess and record the pregnancy outcomes (maternal and neonatal outcomes). Maternal outcomes included placental abruption and postpartum hemorrhage (500 mL after vaginal delivery or 1000 mL after cesarean section). Neonatal outcomes included preterm birth (<37 weeks), SGA (birth weight less than the 10th percentile of birth weight for the same gestational age), FGR, intrauterine distress, neonatal asphyxia, Apgar score <7 within 5 min, and perinatal death. Those who experienced any of the above adverse outcomes were included in the adverse outcome group. Those without any of these adverse pregnancy outcomes were included in the good outcome group. Since cesarean section is the most common consequence of HDP, it was not included as an adverse outcome in this section.

Diagnosis was made according to the Guidelines for the Diagnosis and treatment of hypertension and pre‐eclampsia in pregnancy: a clinical practice guideline in China (2020),^24^ and the diagnostic criteria were in accordance with the 2021 International Society for the Study of Hypertension in Pregnancy classification, diagnosis and management recommendations for international practice.^25^ HDP patients were classified into GH (*n* = 65), CH (*n* = 13), PE‐EC (*n* = 74), and CH with PE (*n* = 4). Since the International Society for the Study of Hypertension in Pregnancy no longer differentiates the severity of PE, this study does not differentiate the severity of patients with PE. The diagnostic criteria regarding this study are as follows:

*GH*: first occurrence of hypertension after 20 weeks of gestation in the absence of proteinuria or other findings suggestive of PE. The average of two measurements taken at least 6 h apart. Hypertension should be defined as a systolic blood pressure (SBP) ≥ 140 mmHg and/or a diastolic blood pressure (DBP) ≥ 90 mmHg. GH returns to normal within 12 weeks postpartum.

*CH*: pre‐existing hypertension or SBP ≥140 mmHg and/or DBP ≥90 mmHg before 20 weeks of gestation without significant exacerbation or manifestation of acute severe hypertension during pregnancy.

PE: maternal SBP ≥140 mmHg and/or DBP ≥90 mmHg after 20 weeks of gestation accompanied by one or more of the following criteria: (1) Proteinuria (urine creatinine ≥30 mg/mmol, or ≥0.3 g/24 h in 24‐h urine sample, or ≥2+ on a urine protein test strip in 24‐h urine sample). (2) Other maternal organ dysfunction, including neurological complications, pulmonary edema hematologic complications, acute kidney injury, liver involvement, hematologic abnormalities, intra‐microvascular hemolysis, and cardiac failure. (3) Uteroplacental dysfunction including FGR, angiogenic imbalance, abnormal umbilical artery by Doppler waveform analysis, placental abruption, or intrauterine fetal death.

*Early‐onset and late‐onset PE*: gestational week at the time of diagnosis of PE, early‐onset (<34 weeks), or late‐onset (≥34 weeks). Tonic convulsions occurring on the basis of PE that cannot be explained by other causes can occur before, during, or after labor, or in the absence of clinical signs of PE.

*CH with PE*: No proteinuria before 20 weeks of gestation and symptoms associated with PE after 20 weeks of gestation; or proteinuria before 20 weeks of gestation and a significant increase in the amount of urinary protein after 20 weeks of gestation.

### 2.3 General clinical information

Patient's clinical history was collected including age, primiparity, assisted reproduction, family history of hypertension, family history of HDP, and classification of HDP.

### 2.4 Laboratory measurements

The study subjects were all pregnant at 20 weeks of gestation range (18 + 1 weeks to 24 + 6 weeks), and 5 mL of fasting elbow vein blood was collected from the pregnant women using a sterile syringe and centrifuged for 30 min at 15,000 rpm at 4°C, and the supernatant was dispensed into pre‐cooled sterile tubes. The samples were frozen at −80°C. PlGF and sFlt‐1 concentrations were measured by electrochemiluminescence immunoassay (Roche Diagnostics, Mannheim, Germany) on a Cobas e601 automated immunoassay analyzer (Roche Diagnostics). PROK1 (Thermo Scientific, MA, USA) levels were determined by enzyme immunoassay. The intra‐assay coefficients of variation for sFlt‐1, PlGF, and PROK1 were less than 2%, 5%, and 10%, respectively, and the inter‐assay coefficients were less than 4.5%, 5%, and 12%, respectively.

### 2.5 Data and statistics

G Power software version 3.17 (G*Power 3.1.9.2, Kiel, Germany) was used to calculate the sample size (significance level of *α* = 0.05, power of 1 − *β* = 0.8, and effect size of *d* = 0.5). The valid sample consisted of more than 102 women. Data were collected and then analyzed using the SPSS 22.0. The Shapiro Wilk test was used to determine the normality of data. Categorical variables were expressed as numbers and percentages (%), and the measurement data were expressed as mean (standard deviation) or median (IOR, interquartile range). Person's chi‐square or Fisher's exact test was used to compare qualitative variables. Kruskal–Wallis H test with post hoc Dunnett's multiple comparisons test was used for more than multiple groups and the results were shown as post hoc test *p*‐value. The predictive utility of the sFlt‐1/PlGF ratio for adverse outcomes in patients with HDP cohorts was assessed using receiver operating characteristic curves (ROC) and the area under the curve (AUC). Differences in AUC between groups were compared using MedCalc 14.12.0 (MedCalc; MedCalc Software Co. Ltd., Ostend, Belgium). *p*‐values <0.05 were considered significant.

## 3 RESULTS

### 3.1 General data

From January 2022 to December 2023, 156 women were finally screened for the study. Out of these 156 patients, 54 (54/156, 34.62%) had adverse outcomes and 102 (102/156, 65.38%) had good outcomes; 41.67% were diagnosed with GH, 8.33% with CH, 47.44% with PE‐EC, and 2.56% with CH with PE. The general information of the patients is shown in Table 1. More than half (68.63%, 74/102) of those with a good outcome were in the appropriate age range (25–34 years), while nearly half (42.59%, 25/54) of those with a an adverse outcome were of advanced maternal age. There was a statistical difference between the two groups in terms of the number of patients with advanced maternal age (*p* < 0.001). Notably, 59.26% of the 54 patients in the adverse outcome group were diagnosed with PE‐EC and 7.41% were diagnosed with CH with PE, which was significantly higher than those in the good outcome group (all *p* < 0.05). In addition, we observed statistically no significant differences between the two groups in terms of assisted reproduction, family history of hypertension or HDP, and mode of delivery (all *p* > 0.05). On the other hand, the number of patients with CH with PE was small, so those patients were merged with patients with PE‐EC in subsequent analyses. For patients with different types of HDP, we also analyzed the general data of these patients, as shown in Table 2, and did not observe any statistical differences in these data between the groups (all *p* > 0.05).

**TABLE 1:** General information on HDP patients with adverse outcomes and good outcomes.

	Adverse outcome	Good outcome	*p*‐value
	(*n* = 54)	(*n* = 102)	
Age (year), (*n*, %)			0.001
18–24	6 (11.11)	9 (8.82)	0.645
25–34	23 (42.59)	74 (68.63)	<0.001
≥35 and <45	25 (46.30)	19 (22.55)	<0.001
Primiparity (*n*, %)	27 (50.00)	42 (41.18)	0.291
Assisted reproduction (*n*, %)	2 (3.70)	1 (0.98)	0.239
Family history of hypertension or HDP history	5 (46.30)	6 (5.88)	0.433
Classification of HDP			0.002
GH	16 (29.63)	49 (48.04)	0.026
CH	2 (3.70)	11 (10.78)	0.128
PE‐EC	32 (59.26)	42 (41.18)	0.031
CH with PE	4 (7.41)	0 (0.00)	0.005
Mode of delivery			
Cesarean section	40 (74.07)	68 (66.67)	0.340
Vaginal delivery	14 (25.93)	34 (33.33)	

*Note*: All data are shown as *N* (%). Person's chi‐square test or Fisher's exact test was used for between‐group comparisons. *p*‐value <0.05 was statistically significant.

Abbreviations: CH, chronic hypertension; CH with PE, chronic hypertension with preeclampsia; GH, gestational hypertension; PE‐EC preeclampsia‐eclampsia.

**TABLE 2:** General information on patients with different types of HDP.

	GH	CH	PE‐EC/CH with PE	*p*‐value
	(*n* = 65)	(*n* = 13)	(*n* = 78)	
Age (year), (*n*, %)				0.432
18–24	5 (10.77)	1 (7.69)	7 (8.97)	0.916
25–34	33 (50.77)	9 (69.23)	51 (65.38)	0.158
≥35 and <45	25 (38.46)	3 (23.08)	20 (25.64)	0.235
Primiparity (*n*, %)	35 (53.85)	6 (46.15)	28 (35.90)	0.098
Assisted reproduction (*n*, %)	1 (1.54)	0 (0.00)	2 (2.56)	1.000
Family history	3 (4.65)	2 (15.38)	6 (7.69)	0.150
Mode of delivery				
Cesarean section	40 (64.54)	8 (61.54)	60 (76.92)	0.136
Vaginal delivery	25 (38.46)	5 (38.46)	18 (23.08)	

All data are shown as *N* (%). Person's chi‐square test or Fisher's exact test was used for between‐group comparisons. *p*‐value <0.05 was statistically significant.

Abbreviations: CH, chronic hypertension; CH with PE, chronic hypertension with preeclampsia; GH, gestational hypertension; PE‐EC preeclampsia‐eclampsia.

### 3.2 Plasma sFlt‐1 and PlGF levels and sFlt‐1/PlGF ratio in patients with HDP

**FIGURE 1:**
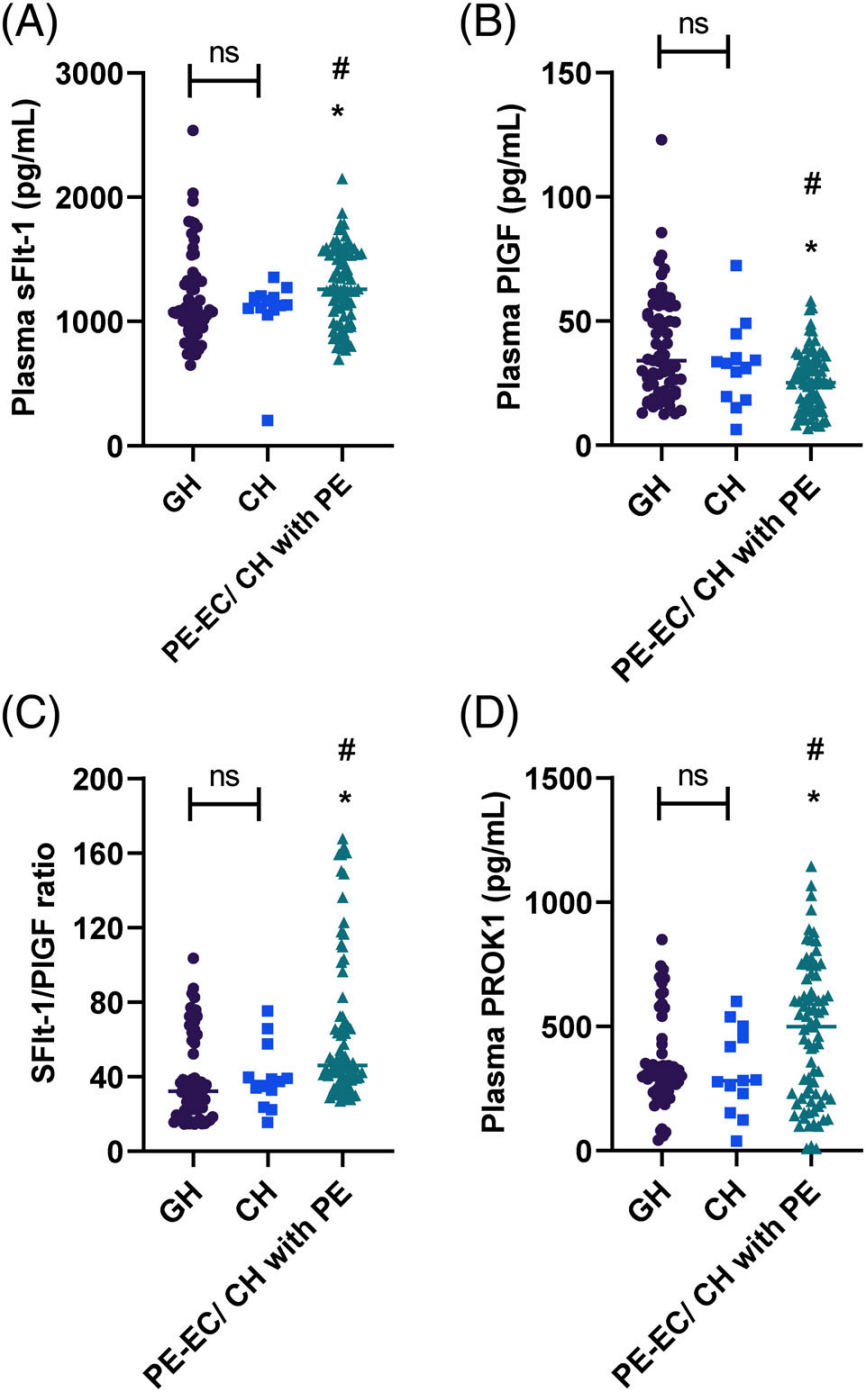
Plasma sFlt‐1, PlGF levels, and sFlt‐1/PlGF ratio in patients with HDP. (A) Plasma sFlt‐1, (B) plasma PlGF, (C) sFlt‐1/PlGF ratio, and (D) plasma PROK1 versus GH *p <0.05; versus CH # *p* <0.05; ns, *p* >0.05.

**FIGURE 2:**
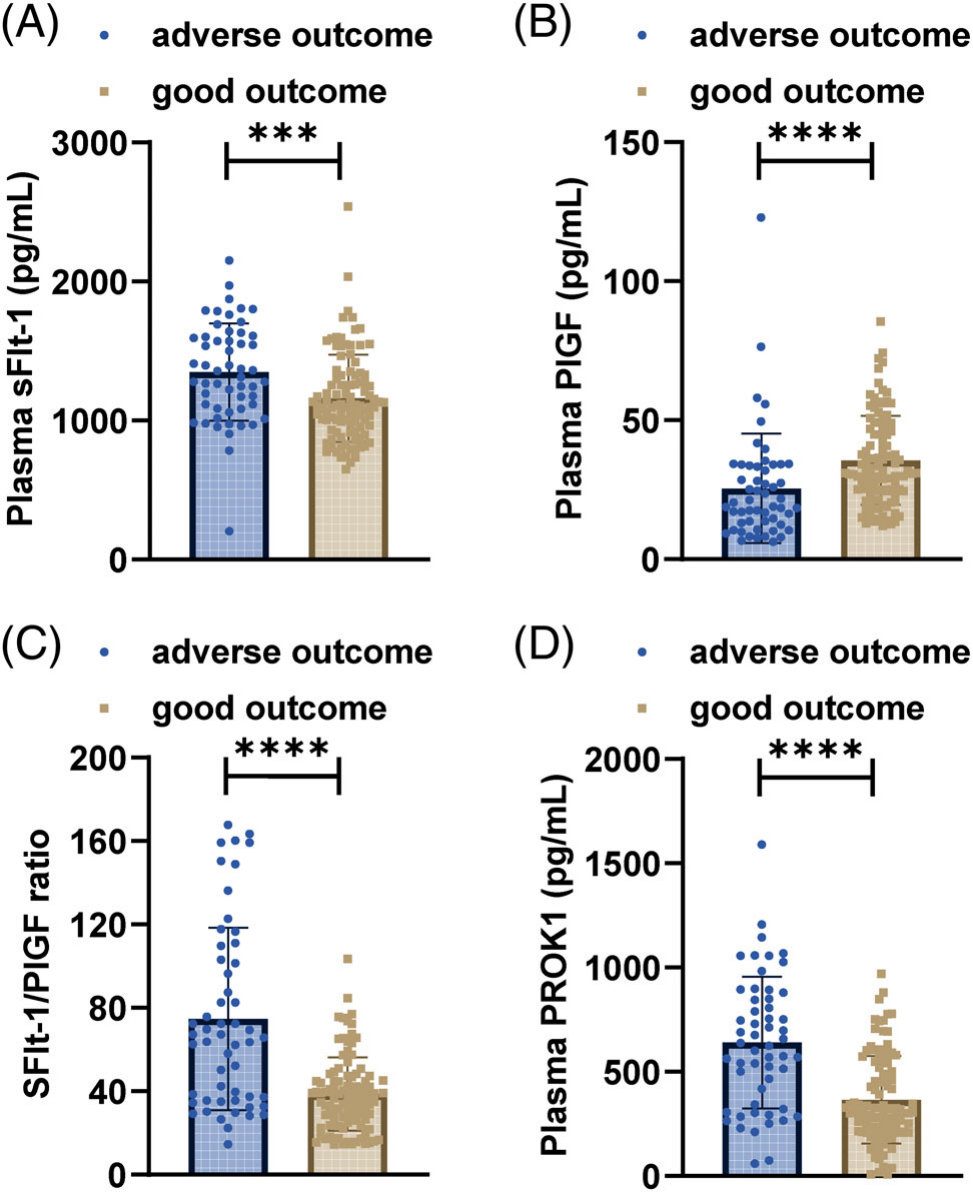
Comparison of plasma sFlt‐1 and PlGF levels and sFlt‐1/PlGF ratio between patients with adverse outcomes and those with good pregnancy outcomes. (A) Plasma sFlt‐1, (B) plasma PlGF, (C) sFlt‐1/PlGF ratio, and (D) plasma PROK1. ***p <0.001, ****p <0.0001.

Plasma marker levels were compared in patients, as shown in Figure 1 and Table 3. Patients with PE‐EC/CH with PE had higher plasma sFlt‐1 levels (Figure 1A, *p*<0.05), lower plasma PlGF levels (Figure 1B, *p*<0.05), higher sFlt‐1/PlGF ratio, and higher plasma PROK1 levels compared with the GH group and CH group (Figure 1C, D, all *p* <0.05). None of these indices were statistically different in the GH group and the CH group (all *p* >0.05). Pregnant women with adverse outcomes exhibited significantly higher sFlt‐1 levels (Figure 2A, *p*<0.001), lower PlGF concentrations (Figure 2B, *p*<0.0001), higher sFlt‐1/PlGF ratio, and higher plasma PROK1 levels (Figure 2C, D, *p*<0.0001) (Figure 2).

**TABLE 3:** Blood biomarker levels in HDP patients.

Indicates	GH (*n* = 65)	CH (*n* = 13)	PE‐EC/CH with PE (*n* = 78)	*p*‐value
sFlt‐1 (pg/mL)	1077.81 [978.61, 1322.63]	1094.12 ± 268.25	1259.32 [1004.86, 1547.04]	0.035
PIGF (pg/mL)	34.00 [21.87, 52.99]	32.87 [19.56, 35.19]	25.88 ± 12.11	<0.001
SFlt‐1/PlGF ratio	32.30 [21.99, 58.00]	39.70 ± 16.48	46.12 [39.24, 68.85]	<0.001
PROK1 (pg/mL)	300.77 [250.00, 345.29]	319.94 ± 163.93	498.84 [213.67, 678.30]	0.027

*Note*: Normal distribution represented as mean (SD), while nonparametric represented as median (IQR). Use Kruskal Wallis H test to compare the differences among the three groups.

### 3.3 Adverse maternal outcomes and neonatal outcomes in patients with HDP

Next, we analyzed the adverse outcomes in patients with HDP, as shown in Table 4. The results showed that, with the exception of preterm labor (*p* = 0.002), there were no significant differences in adverse maternal outcomes and neonatal outcomes between patients (all *p* >0.05). The number of adverse pregnancy outcome events in patients with PE‐EC/CH with PE was as high as 46.15% (36/78) (*p* <0.001) (Multiple adverse pregnancy outcomes in the same patient were also defined as one event), which was significantly higher than that in the GH group (24.62%, 16/65) and CH group (15.38%, 2/13).

**TABLE 4:** Adverse pregnancy outcomes in patients with HDP.

Outcome		HDP final diagnosis			
	All HDP	GH	CH	PE‐EC/CH with PE (*n* = 78)	*p*‐value
	(*n* = 156)	(*n* = 65)	(*n* = 13)		
Maternal outcome					
Placental abruption	2 (1.28)	0 (0.00)	0 (0.00)	2 (2.56)	0.581
Postpartum hemorrhage	1 (0.64)	0 (0.00)	0 (0.00)	1 (1.28)	1.00
Neonatal outcomes					
Premature	44 (28.21)	10 (15.38)	1 (7.69)	30 (38.46)	0.002
SGA	14 (8.97)	3 (4.62)	0 (0.00)	11 (14.10)	0.100
FGR	4 (2.56)	1 (1.54)	0 (0.00)	3 (3.85)	0.737
Fetal intrauterine distress	4 (2.56)	0 (0.00)	1 (7.69)	3 (3.85)	0.129
Apgar 5 min <7	5 (3.21)	3 (4.62)	1 (7.69)	3 (3.85)	0.595
Perinatal death	1 (0.64)	0 (0.00)	0 (0.00)	1 (1.28)	1.00
All outcomes	54 (34.62)	16 (24.62)	2 (15.38)	36 (46.15)	0.008

*Note*: All data are shown as *N* (%). Categorical values were performed using Fisher's exact test. *p*‐value <0.05 was statistically significant.

Abbreviations: FGR, fetal growth; SGA, small for gestational age.

### 3.4 Predictive value of sFlt‐1/PlGF ratio in adverse pregnancy outcomes in patients with HDP

**FIGURE 3:**
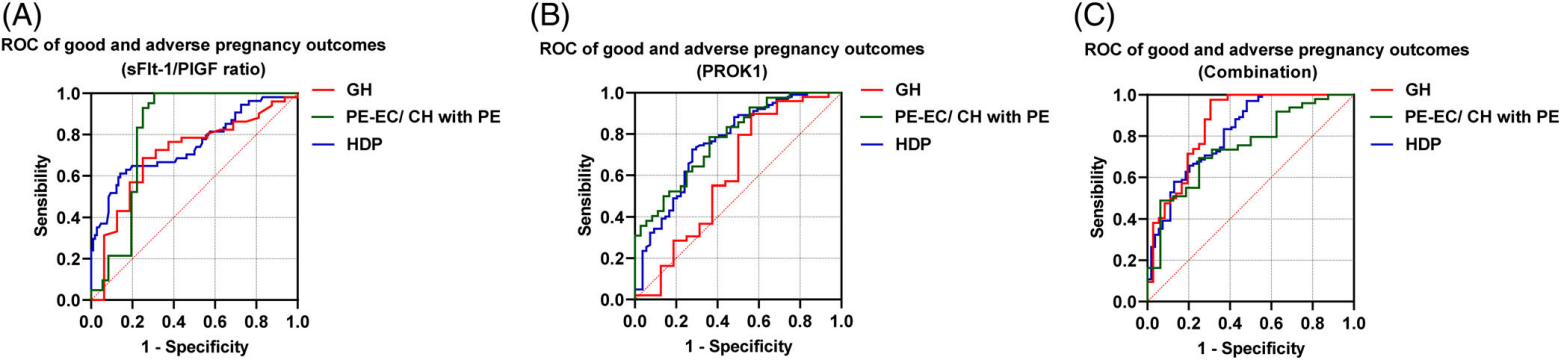
Predictive value of ROC curves and area under the curve for adverse pregnancy outcomes in patients with HDP. (A) sFlt‐1/PlGF ratio, (B) PROK1, (C) sFlt‐1/PlGF ratio combined with PROK1.

We illustrated the efficacy of adverse pregnancy outcomes in patients with HDP by plotting ROC curves and calculating AUC, as shown in Table 5 and Figure 3. Due to the small number of patients with CH, we did not analyze this group of patients separately. The results showed that the sFlt‐1/PlGF ratio (Figure 3A), plasma PROK1 (Figure 3B), and the combined metrics (Figure 3C) had high predictive value for adverse outcomes in patients in the PE‐EC/CH with PE group as compared with the GH group (all *p* <0.05, 0.706 vs. 0.813, 0.610 vs. 0.610 vs. 0.778, 0.751 vs. 0.860). No statistically significant difference was found in the AUC of sFlt‐1/PlGF ratio and plasma PROK1 in the PE‐EC/CH with PE group and total HDP patient cohort (both *p* >0.05, 0.813 vs. 0.778, 0.759 vs. 0.751); however, sFlt‐1/PlGF ratio in combination with plasma PROK1 improved the diagnosis of adverse pregnancy outcomes in the PE‐EC/CH with PE group and the total HDP cohort, respectively, as a separate metric (both *p* <0.05, 0.860 vs. 0.813 and 0.778, 0.817 vs. 0.759 and 0.751). In addition, no predictive value of plasma PROK1 for adverse pregnancy outcomes was observed in patients with GH.

**TABLE 5:** The value of analyzing blood indicators for good or adverse pregnancy outcomes in different types of HDP cohorts for parturients.

HDP cohorts	Indicators	Cut off	AUC (95%CI)	*p*‐value	Sensitivity	Specificity
GH	sFlt‐1/PlGF ratio	35.45	0.706 (0.560–0.853)	0.013	70.83	77.00
PE‐EC/CH with PE		67.64	0.813 (0.700–0.927)	<0.001	91.62	66.89
HDP		58.83	0.759 (0.677–0.841)	<0.001	62.12	87.85
GH	PROK1	495.92	0.610 (0.425–0.794)	0.190	89.8	43.75
PE‐EC/CH with PE		622.65	0.778 (0.678–0.879)	<0.001	80.57	65.89
HDP		462.6	0.759 (0.677–0.841)	<0.001	75.55	75.52
GH	Combination	/	0.751 (0.618–0.884)	0.003	71.39	77
PE‐EC/CH with PE		/	0.860 (0.775–0.946)	<0.001	97.62	72.44
HDP		/	0.817 (0.746–0.888)	<0.001	97.06	54.85

*Note*: AUC, area under curve; CH with PE, chronic hypertension with preeclampsia; GH, gestational hypertension; HDP, hypertensive disease of pregnancy; PE‐EC preeclampsia‐eclampsia; 95%CI, confidence interval. *p‐*value <0.05 was statistically significant.

## 4 DISCUSSION

HDP is associated with significant adverse pregnancy outcomes.^26^ The major findings of this study include:(1) 34.62% (54/156) of all HDP patients showed at least one adverse pregnancy outcome, with the PE‐EC/CH with PE group showing the highest incidence of adverse outcomes (46.15); (2) Compared to the GH group, patients in the PE‐EC/CH with PE group had higher plasma sFlt‐1 /PlGF ratio and PROK1 levels; (3) sFlt‐1/PlGF ratio, plasma PROK1, and combined metrics have good predictive values in predicting adverse pregnancy outcomes in patients with HDP, especially in the PE‐EC/CH with PE cohort. Among HDP patients in this study, 47.44% were diagnosed with PE; of these, the preterm birth rate in women with PE‐EC was 38.46%. In our study, it was observed that PE was also associated with adverse perinatal outcomes in neonates with SGA. In HDP patients, early neonatal birth remains a significant contributor to adverse pregnancy outcomes, which necessitates improved treatment approaches.

To date, research on the etiologic mechanisms of HDP has been poorly defined, and there is a lack of effective clinical tools for targeted prevention. In recent years, the relationship between inflammation and HDP has become a key focus in the etiologic study of HDP. It has been found that the process of pregnancy itself involves a mild inflammatory response process, and HDP is associated with placental, inflammatory, and progressive endothelial damage, with markedly elevated levels of multiple inflammatory factors and vascular endothelial disorders causing a wide range of maternal symptoms.^27^ Animal tests have also confirmed the correlation between inflammation and GH.^28^ In terms of inflammatory indicators, such as C‐reactive protein and tumor necrosis factor‐α, although they are more sensitive in diagnosing HDP, they overlap with more diseases and are easily affected by the pregnant woman's own inflammatory response or other factors. The interplay of factors promoting and inhibiting angiogenesis in the placenta and maternal blood flow has emerged as a central topic in both national and global studies on HDP. Factors related to vascular endothelial cells are mainly PlGF and sFlt‐1, of which the sFlt‐1/PlGF ratio has been clinically utilized as an emerging indicator for predicting PE in patients with HDP. Several studies have already demonstrated the value of the sFlt‐1/PlGF ratio in predicting PE severity and adverse pregnancy outcomes.^29^, ^30^ HDP is a common complication in obstetrics, and these disorders may lead to maternal and perinatal deaths, PE‐EC, in particular, poses a serious threat to the health and safety of mothers and infants.^31^ Its pathophysiological process involves various aspects such as placental vascular dysfunction, endothelial cell injury, and inflammatory response. sFlt‐1 is one of the receptors for vascular endothelial growth factor, which regulates angiogenesis and vascular permeability. PlGF‐1 is a protein secreted by the placenta, which promotes angiogenesis and maintains placental perfusion. The dysfunction of the dynamic balance of placental angiogenesis caused by the increase of sFlt‐1 and the decrease of PlGF leads to insufficient invasion of the endometrium by trophoblasts, thus impeding the reconditioning of the uterine spiral arteries, which ultimately leads to ischemia and hypoxia of the placenta and thus to a wide range of adverse pregnancy outcomes. Elevated sFlt‐1 levels in late pregnancy are associated with adverse pregnancy outcomes such as preterm labor, low birth weight, and respiratory distress syndrome.^32^ It is well known that PE is a severe condition of HDP, which is combined with damage to the heart, brain, lungs, and other organs and is accompanied by fetal dysplasia.^31^ In this study, we first observed 54 adverse pregnancy outcomes in HDP patients, of which 66.67% were PE‐EC/CH with PE. Patients with PE‐EC/CH with PE had a higher sFlt‐1/PlGF ratio than patients with CH and GH.

PROK1 is characterized as a factor that promotes angiogenesis, ensuring maximum blood vessel flow during pregnancy.^33^ Endothelial damage caused by HDP affects the oxygen supply to the placenta. Many angiogenic factors are regulated by this physiologic hypoxia, including PROK1.^34^ PROK1 is upregulated in hypoxia, suggesting that it may contribute to angiogenesis.^35^, ^36^ Mechanistically, elevated PROK1 may reduce blood flow and oxygen supply to the placenta by affecting VEGF contraction, which in turn increases the risk of intrauterine growth restriction, fetal distress, neonatal asphyxia, and other adverse pregnancy outcomes.^37^ In the present study, we also observed higher plasma PROK1 in patients with PE‐EC/CH with PE than in patients with CH and GH. Low maternal concentrations of PIGF and high concentrations of sFlt‐1 are detected in SGA fetuses.^37^ Elevated levels of PROK1 in amniotic fluid at mid‐pregnancy predict adverse pregnancy outcomes.^23^ This study listed adverse pregnancy outcomes in patients with each type of HDP and only found differences in preterm labor rate, although this cannot be ruled out due to the small sample size. sFlt‐1/PlGF ratio and combined index (sFlt‐1/PlGF ratio combined with plasma PROK1) were effective in differentiating between adverse and good pregnancy outcomes in HDP cohorts; however, plasma PROK1 was not effective in differentiating between adverse and good pregnancy outcomes in this cohort. The reasons for this need to be confirmed by more relevant studies. Of interest, the sFlt‐1/PlGF ratio, plasma PROK1, and combined metrics were effective in differentiating between adverse and good pregnancy outcomes in the PE‐EC/CH with PE cohort. In addition, these metrics had good discriminatory efficacy in the total HDP cohort. sFlt‐1/PlGF ratio, plasma PROK1, and combined metrics had discriminatory efficacy for pregnancy outcomes in the total HDP cohort in part due to the effect of the PE‐EC/CH with PE cohort. Thus, the predictive value of these indicators in the GH cohort was lower than in the PE‐EC/CH with PE cohort. Finally, the combined metrics improved the predictive value of the individual metrics for adverse pregnancy outcomes in the PE‐EC/CH with PE cohort and the total HDP cohort, respectively.

## 5 LIMITATIONS

This study was a single‐center study with small sample size, and no correction factors were used for the effect of adverse pregnancy outcomes in HDP cohorts, which biased the results and may limit the generalizability of the results. The present study was a cross‐sectional cohort study, which only establishes associations and not causality. Additional studies using correlates associated with pathologic placenta can provide a causal relationship between factors and adverse outcomes. Although we used a combined metric (sFlt‐1/PlGF ratio combined with plasma PROK1) to assess adverse pregnancy outcomes in multiple cohorts, however, we did not explore a strong correlation between the two, and more correlation studies are needed to confirm this.

## 6 CONCLUSION

sFlt‐1/PlGF ratio and plasma PROK1 have been shown to predict adverse pregnancy outcomes in HDP cohorts and in the PE‐EC/CH with PE cohort. In addition, the combined metrics improve the efficacy of predicting adverse pregnancy outcomes in HDP cohorts. It provides a clinical basis for predicting and preventing adverse pregnancy events in patients with HDP.

## CONFLICT OF INTEREST STATEMENT

The authors have no conflicts of interest to declare.

## ETHICS STATEMENT

All procedures performed in this study involving human participants were in accordance with the ethical standards of the institutional and/or national research committee and with the 1964 Helsinki Declaration and its later amendments or comparable ethical standards. All subjects were approved by Shengzhou People's Hospital (No.202101SZ‐05).

## Data Availability

Data is available from the corresponding author on request.
